# I-*kappa*-kinase-2 (IKK-2) inhibition potentiates vincristine cytotoxicity in non-Hodgkin's lymphoma

**DOI:** 10.1186/1476-4598-9-228

**Published:** 2010-09-01

**Authors:** Ayad Al-Katib, Alan A Arnold, Amro Aboukameel, Angela Sosin, Peter Smith, Anwar N Mohamed, Frances W Beck, Ramzi M Mohammad

**Affiliations:** 1 Lymphoma Research Laboratory, Wayne State University - School of Med, 540 E. Canfield, 8229 Scott Hall, Detroit, MI 48201, USA; 2 Fox Chase Cancer Center, 333 Cottman Avenue, Philadelphia, PA 19111-2497, USA; 3 Millennium Pharmaceuticals, 40 Landsdowne Street, Cambridge, Massachusetts 02139, USA; 4 Clinical Pathology, Wayne State University School of Medicine, 4727 St. Antoine, Detroit, MI 48201, USA; 5 Internal Medicine, Wayne State Univeristy, 2224 Ellimann Bldg, 421 E. Canfield, Detroit, MI 48201, USA; 6 Karmanos Cancer Institute, 732 HWCRC, 4100 John R, Detroit, MI 48201, USA

## Abstract

**Background:**

IKK-2 is an important regulator of the nuclear factor-κB (NF-κB) which has been implicated in survival, proliferation and apoptosis resistance of lymphoma cells. In this study, we investigated whether inhibition of IKK-2 impacts cell growth or cytotoxicity of selected conventional chemotherapeutic agents in non-Hodgkin's lymphoma.

Two established model systems were used; Follicular (WSU-FSCCL) and Diffuse Large Cell (WSU-DLCL2) Lymphoma, both of which constitutively express p-IκB. A novel, selective small molecule inhibitor of IKK-2, ML120B (*N*-[6-chloro-*7*-methoxy-*9H*-β-carbolin-8-yl]-2-methylnicotinamide) was used to perturb NF-κB in lymphoma cells. The growth inhibitory effect of ML120B (M) alone and in combination with cyclophosphamide monohydrate (C), doxorubicin (H) or vincristine (V) was evaluated *in vitro *using short-term culture assay. We also determined efficacy of the combination *in vivo *using the SCID mouse xenografts.

**Results:**

ML120B down-regulated p-IκBα protein expression in a concentration dependent manner, caused growth inhibition, increased G0/G1 cells, but did not induce apoptosis. There was no significant enhancement of cell kill in the M/C or M/H combination. However, there was strong synergy in the M/V combination where the vincristine concentration can be lowered by a hundred fold in the combination for comparable G2/M arrest and apoptosis. ML120B prevented vincristine-induced nuclear translocation of p65 subunit of NF-κB. *In vivo*, ML120B was effective by itself and enhanced CHOP anti-tumor activity significantly (P = 0.001) in the WSU-DLCL2-SCID model but did not prevent CNS lymphoma in the WSU-FSCCL-SCID model.

**Conclusions:**

For the first time, this study demonstrates that perturbation of IKK-2 by ML120B leads to synergistic enhancement of vincristine cytotoxicity in lymphoma. These results suggest that disruption of the NF-κB pathway is a useful adjunct to cytotoxic chemotherapy in lymphoma.

## Background

NHL is the fifth most common type of cancer in the US representing 4.5% of cancer cases. Since the early 1970's the incidence of NHL has doubled [1]. It is a group of heterogeneous diseases resulting from malignant transformation of lymphocytes. Eighty-five percent of NHLs are B-cells that can be broadly classified as aggressive (50%) and indolent (40%). Diffuse Large B-cell NHL (DLBCL) is the most common subtype (30%) of all lymphomas and is the prototype of aggressive but curable NHL. Follicular lymphoma (FL) is the second most common subtype, representing 22% and is the most common indolent NHL [2,3].

To date, there is no consensus concerning the best treatment algorithm, but combination chemotherapy has been the mainstay for treatment of NHL. Incorporation of the anti-CD20 monoclonal antibody, Rituximab, has led to improvements in overall survival [4,5]. More than half of patients with DLBCL can be cured with combination of Rituximab (R) and cyclophosphamide, doxorubicin, vincristine and prednisone (CHOP). Incorporating Rituximab into conventional chemotherapy for follicular lymphoma has lead to higher response rates and longer durations of remission compared with chemotherapy alone [6]. The success of Rituximab suggests that additional targeted therapeutics might improve the efficacy of cytotoxic regimens.

Constitutively active NF-κB in lymphoma is known to induce resistance to intrinsic and extrinsic apoptosis pathways [7]. NF-κB is a transcription factor comprised of homo- and heterodimers, p50/p105 (NF-κB1), p52/p100 (NF-κB2), c-Rel, RelB, and p65 (RelA) [8]. Inhibitors of kappa B (IκBα, IκBβ and IκBε) contain ankyrin-like repeats that mediate sequestration of NF-κB in the cytosol [9]. The interaction between IκBα and NF-κB is regulated by IκB kinase (IKK-1 and IKK-2). Phosphorylation of IκBα leads to its degradation and release of NF-κB. NF-κB is then able to translocate to the nucleus where it controls a number of molecules involved in vital cellular functions, such as proliferation, apoptosis, and resistance to chemotherapy [10-16].

Clinically, aberrant NF-κB activation has been linked to poor outcome in lymphomas [17,18]. Therefore, these and other studies prompted us to investigate potential therapeutic effects of inhibiting components of the NF-κB activation pathway in our lymphoma models.

Small molecule inhibitors (SMI) are used to selectively target molecules involved in survival pathways. ML120B (*N*-[6-chloro-*7*-methoxy-*9H*-β-carbolin-8-yl]-2-methylnicotinamide) is a potent and selective inhibitor of IKK-2, acting through blockade of the ATP-binding site in the kinase. ML120B has been shown to inhibit tumor necrosis factor-α (TNF-α)-induced nuclear translocation of p65 subunit of NF-κB and block TNF-α-stimulated cytokine production in human fibroblast-like synovial cell cultures isolated from patients with rheumatoid arthritis [19]. ML120B inhibits both baseline and TNF-α-induced NF-κB activation in multiple myeloma cells. It was also shown to inhibit the growth of multiple myeloma cells *in vitro *and *in vivo *SCID mouse models [20].

In this report, we show that ML120B inhibits the phosphorylation of IκBα, hinders the growth of lymphoma cell lines in a concentration- and time-dependent manner and reduces progression out of G0/G1 phase of the cell cycle. More importantly, ML120B has a synergistic interaction with vincristine, a common cytotoxic agent used in the treatment of hematological malignancies. Our studies suggest that IKK-2 inhibition has a therapeutic role in lymphoma when used alone or in combination with cytotoxic agents.

## Results

### Inhibition of IKK-2 Leads to Growth Inhibition of Lymphoma Cell Lines

**Figure 1:**
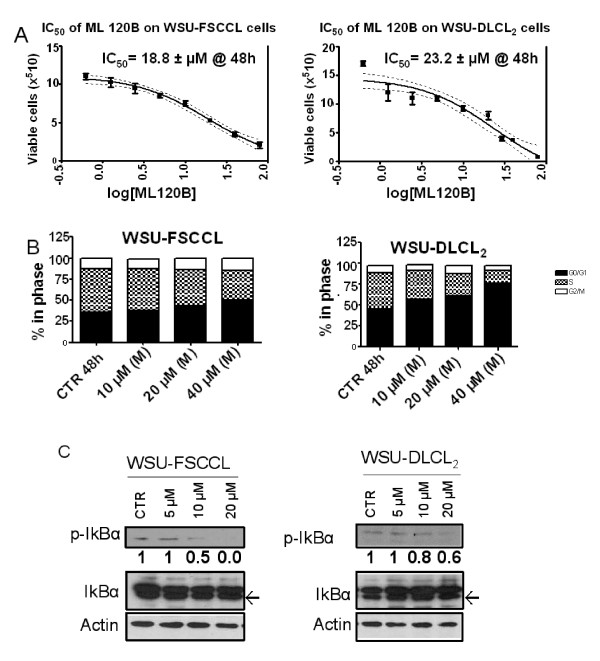
**ML120B inhibits cell growth, cell cycle progression and phosphorylation of IκBα in Lymphoma cell lines**. A. IC_50 _was calculated by trypan blue exclusion assay for each cell line after 48 h incubation. Cell number (vertical axis) = Mean and SEM [standard error of mean]. B. DNA content was analyzed by PI staining using flow cytometry after 48 h for each cell line. C. Lymphoma cell lines were cultured for 60 minutes with ML120B at the indicated concentrations and whole cell lysates were subjected to Western blot for detection of p-IκBα, IκBα and actin (Arrows indicate total IκBα).

In order to determine whether perturbation of the NF-κB activation pathway might play a survival role in our lymphoma models, we seeded lymphoma cells in cluster plates and treated the cells with ML120B at 0 to 80 μM. ML120B inhibited the growth of our cells in a concentration- and time-dependent manner. After 48 hours of incubation, the IC_50 _values were 18.8 and 23.2 μM for WSU-FSCCL and WSU-DLCL_2 _cells; respectively (Figure 1A). Accumulation of cells in sub G0/G1 fraction was not seen, indicating absence of apoptosis (data not shown). Instead, there was a concentration-dependent increase in cells arrested at G0/G1 and a reciprocal decrease of cells in S-phase (Figure 1B). ML120B (40 μM) induced a statistically significant increase of cells in G0/G1: 14% and 31% in WSU-FSCCL and WSU-DLCL_2 _(P = 0.02 and 0.01), respectively.

The WSU-FSCCL and WSU-DLCL_2 _cells exhibit constitutive NF-κB activation as shown by the baseline expression of the phosphorylated form of IκBα (p-IκBα). ML120B inhibited phosphorylated IκBα in a concentration-dependent manner within one hour incubation with these cells. At 20 μM, ML120B inhibited p-IκBα by 100% in WSU-FSCCL and 40% in WSU-DLCL_2 _compared to control (Figure 1C).

### IKK-2 Inhibition in Combination with Conventional Chemotherapeutic Agents

**Figure 2:**
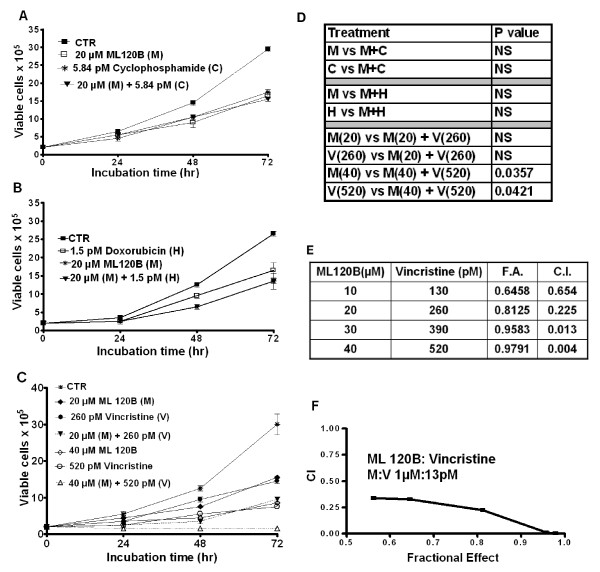
**ML120B synergizes with the microtubule inhibitor, vincristine**. Cell viability was measured by trypan blue exclusion assay over a 72 h incubation period, Mean and standard deviation (SD). WSU-FSCCL cells were exposed to each agent at the indicated concentrations for 72 h or pretreated with ML120B (20 μM) for 60 minutes followed by (A) cyclophosphamide (5.84 pM), (B) doxorubicin (1.5 pM), or (C) vincristine (260 pM, or 520 pM). D. Student t-test was used to calculate the values for combinational treatments. E. Synergy was calculated using CalcuSyn V2; CI < 0.9 indicates synergy. ML120B:vincristine synergized at all concentrations used. F. Combinational index (CI) curve according to Chou-Talalay method (reference 24) used to determine synergy between ML120B and vincristine. A ratio of <0.9 indicates synergy. Abbreviations used: M = ML120B, C = cyclophosphamide monohydrate, H = doxorubicin, V = vincristine, CTR = control (vehicle-treated cells).

Since CHOP is currently the standard regimen for lymphoma therapy, we chose to investigate the effects of IKK-2 inhibition in combination with the cytotoxic components of CHOP in WSU-FSCCL, i.e. cyclophosphamide monohydrate (C), doxorubicin (H), and vincristine (V). The drug concentrations used were adopted from experiments in the WSU-DLCL_2 _cells as previously determined in our lab [21]. When used together, the IC_50 _for WSU-DLCL_2 _were as follows: C = 5.84 pM; H = 1.5 pM; and V = 260 pM. However, when these drugs used individually against WSU-FSCCL in this study, we obtained less than 50% growth inhibitions at 48 hours: C = 38% (i.e. IC_40_); H = 24% (i.e. IC_25_); and V = 39% (i.e. IC_40_). WSU-FSCCL cells were pre-incubated with ML120B for 1 hour at IC_50 _(18.8 μM) prior to addition of cytotoxic agents. The M/C combination did not induce growth inhibition greater than the individual agents alone (Figure 2A & Table insert D). Similar results were obtained with M/H combination (Figure 2B & Table insert D). However, the M/V combination induced significant growth inhibition greater than either agent alone (Figure 2C & Table insert D). To determine if this interaction is synergistic, WSU-FSCCL cells were incubated with both agents at varying concentrations and the fractional effect of both agents alone and in combination was calculated (Figure 2F). The M/V combination at 20 μM: 260 pM yielded a CI value of 0.225 which correlates with "strong synergism". Increasing concentration of the agents yielded "very strong synergism". Notably, M/V combination at lower concentration (10/130) also yielded "synergism" (Table insert E in Figure 2).

### ML120B: Vincristine Combination Induces G2/M Cell Cycle Arrest and Apoptosis

**Figure 3:**
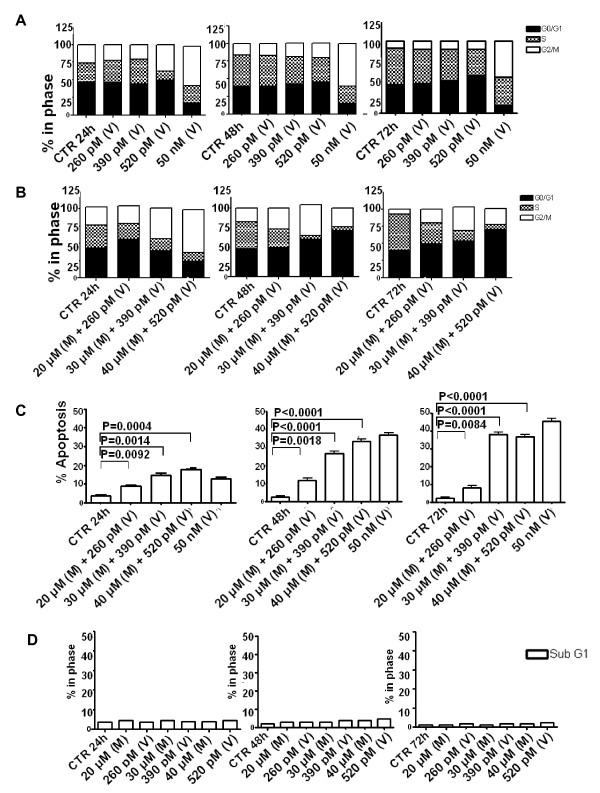
**ML120B:vincristine combination induces cell cycle arrest and apoptosis**. Flow cytometric analysis of cell cycle was done following PI staining of WSU-FSCCL cells. A. WSU-FSCCL cells were exposed to vincristine (V) for the indicated times and concentrations. B. WSU-FSCCL cells were pretreated with ML120B (M) for 60 minutes followed by vincristine (V) at the indicated concentrations and times. C. Apoptotic cells (Sub G0) analyzed by PI staining using flow cytometry in WSU-FSCCL for combinational treatments, Mean and standard error of mean (SEM). D. Apoptotic cells analyzed as in (C) in WSU-FSCCL cells treated with single agents.

**Figure 4:**
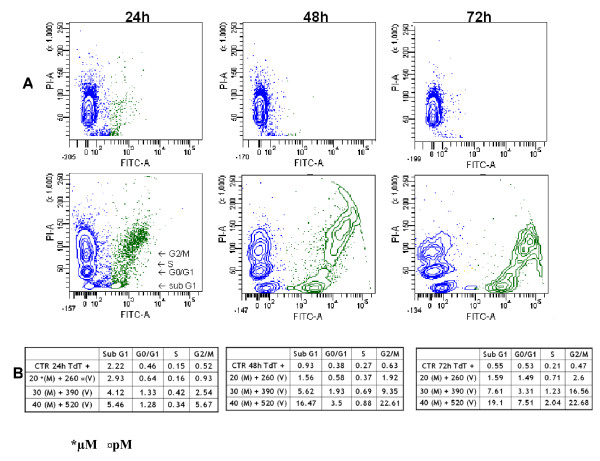
**ML120B:vincristine combination induces apoptosis in G2/M phase cells**. WSU-FSCCL cells were pretreated with ML120B (M) for 60 minutes followed by vincristine (V) at indicated concentrations in Panel B then analyzed for Terminal transferase dUTP Nick End Labeling (TUNEL) staining using flow cytometry. A. Representative histograms using the higher concentrations of M and V; upper panel: untreated, control cells; lower panel: treated with ML120B (40 μM) and vincristine (520 pM). B. Full data of FITC positive cells by cell cycle phase (sub G0, G0/G1, S, G2/M) according to PI staining of DNA. The largest FITC positive population in treated cells was in G2/M followed by sub G0.

**Figure 5:**
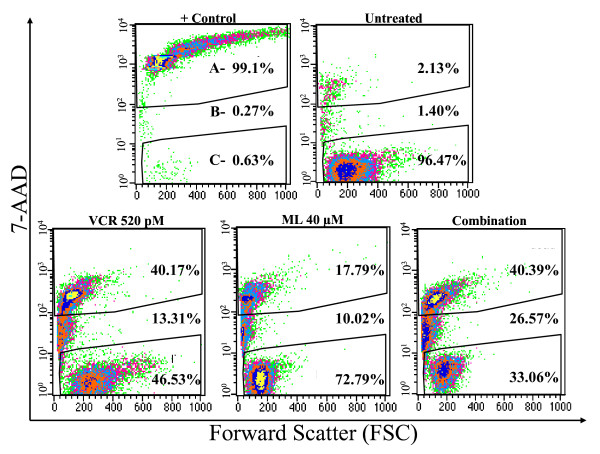
**7-AAD flow cytometric analysis of apoptosis**. Representative scattergrams generated from 7-AAd staining of WSU-FSCCL treated with vincristine at 520 pm (VCR), ML120B at 40 uM (ML) or the combination (ML + VCR) for 72 h (bottom panel left to right). Top panel left (+ control) are heat-treated dead cells; top left (untreated) are live WSU-FSCCL cells from control culture. Within each scattergram, 7-AAD (vertical axis) separates cells into dead (A, top), apoptotic (B, middle) or live (C, bottom).

When used alone against the WSU-FSCCL cells, vincristine at 520 pM induced G2/M arrest at 24 hours which was released at subsequent time points (Figure 3A). Vincristine at its IC_40 _(260 pM) did not induce significant G2/M arrest compared to control. Higher concentrations of vincristine (50 nM to 0.2 μM) were previously shown to induce G2/M arrest in cancer cells [22,23]. Vincristine, at a concentration of 50 nM in our WSU-FSCCL cells induced a sustained G2/M arrest over the 72 hour incubation period (Figure 3A). In the combination studies, the general effects on cell cycle were similar but the magnitude was different depending on the concentration of the 2 compounds. The most dramatic effect was seen with the higher concentrations (M 40 μM: V 520 pM) which showed a significant increase in G2/M at 24 hrs at the expense of S and G0/G1 (Figure 3B). With further incubation (48 and 72 hrs), there was relative increase in G0/G1 suggesting that cells arrested in G2/M at 24 hrs underwent apoptosis. In support of this interpretation is the increase of cells in sub-G0 shown in Figure 3C. The combination of 40:520 M(μM): V(pM) induced a G2/M arrest at 24 hours that was not statistically different from the 50 nM vincristine. The combination of the two agents had a statistically significant concentration- and time-dependent increase in apoptotic sub G0/G1fraction of cells. The increase in the apoptotic fraction induced by the combination was higher compared to that found in both the control and 50 nM vincristine at 24 hours (Figure 3C). At 48 hours, the M/V combination at 40 μM: 520 pM induced comparable apoptosis to the higher concentration of single agent vincristine (50 nM). However, at 72 hours, the high concentration of vincristine alone was more effective. Of special interest is our observation that neither ML120B nor vincristine alone (at concentrations up to 40 μM and 520 pM, respectively) induced sub G0/G1 accumulation (Figure 3D). Therefore, our data suggest that the M/V combination induces an initial G2/M arrest at 24 hours, followed by apoptosis at 48 and 72 hours, leaving a fraction of unaffected cells arrested in G0/G1. We used TUNEL assay to confirm that apoptosis occurred mostly in G2/M. Figure 4A shows the increasing FITC positive population in treated cells compared with control (horizontal axis). Total FITC positive cells also increased with increasing time of incubation and concentration of M:V. For example, the total number of FITC positive cells in the highest concentration of M:V combination (bottom line, panel B, Figure 4) increased from 12.75% at 24 h to 43.46% at 48 h and to 51.33% at 72 h. Moreover, there was progressive shift-to-the-right of FITC positive cell population with increasing incubation indicating increasing intensity of DNA breaks (apoptosis). Most of the FITC positive cells were in G2/M phase followed by sub G0/G1 (which indicates late apoptosis). Some apoptosis did occur from G0/G1 at 72 h. Induction of apoptosis was also confirmed independently using 7-amino-actinomycin D (7-AAD) staining as shown in Figure 5.

### Mechanism of Interaction Between IKK-2 Inhibition and Vincristine

**Figure 6:**
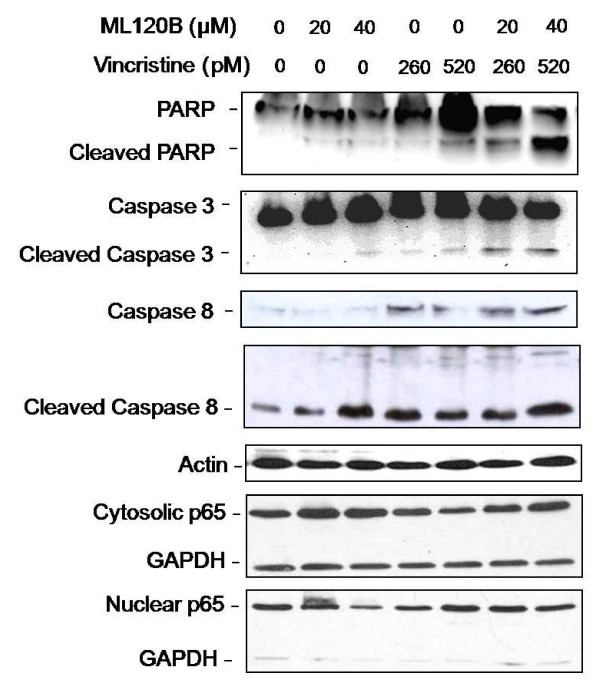
**IKKβ inhibition in combination with Vincristine reduces p65 nuclear translocation and induces apoptosis**. WSU-FSCCL cells were incubated with ML120B or Vincristine alone, or pretreated with ML120B (60 minutes) then cultured with Vincristine at indicated concentrations for 24 h. Total cell lysates were subjected to Western blot for detection of indicated protein using actin as a loading control. Nuclear and cytosolic p65 protein fractions were extracted from total cell lysates. GAPDH is used as a cytosolic loading control.

**Figure 7:**
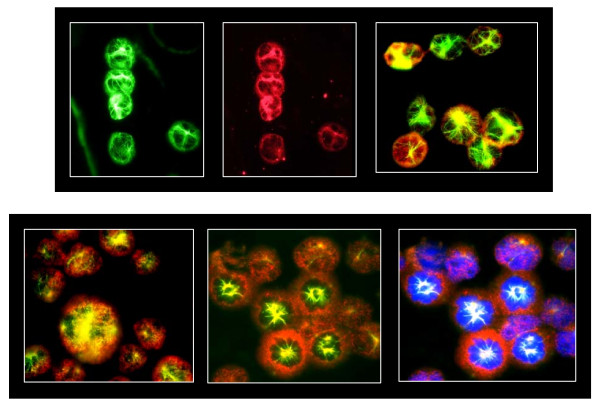
**Immunofluorescence microscopy images of WSU-FSCCL cells**. **Top **panel represents control cells after 48 h of culture; top left, tubulin staining; top center, NF-κB p65; top right, overlay of tubulin and p65. **Bottom **panel represents images of WSU-FSCCL cells after 48 h of treatment with ML120B (40 μM) and vincristine (520 pM). ML120B was added 60 minutes prior to vincristine: Bottom left are vincristine-alone treated cells showing disruption of microtubules and translocation of p65 to the nucleus as indicated by yellow-orange color. Bottom center: ML120B plus vincristine treatment showing sequestration of p65 in the cytoplasm in most cells. Bottom right: is same as bottom center except with DAPI counterstain to demarcate the nuclei and confirm absence of p65 from nuclei in most cells.

To define some of the molecular mechanisms by which ML120B synergizes with vincristine in WSU-FSCCL cells, we first evaluated selected markers of apoptosis. There was only minimal activation of apoptosis executioner (caspase-3) by each agent alone (Figure 6). However, the ML120B: vincristine combination induced caspase-3 cleavage. The combination also significantly induced PARP cleavage. Both agents, individually and in combination, enhanced the expression of cleaved caspase 8. These findings support the flow cytometry data presented in Figures 3, 4, 5 showing that ML120B: vincristine combination induces significant apoptosis whereas neither agent alone has significant effect at the concentrations used in this study. These data also support the synergistic growth inhibitory effect shown in Figure 2. To explain such synergy, we evaluated the effects of different treatments on p65 in WSU-FSCCL cells by western blots and immunofluorescence. As shown in Figure 6, exposure of cells to ML120B led to retention of p65 in the cytosol and reduction in nuclear p65. This finding is consistent with published data [19] and with our finding that ML120B inhibits the phosphorylation, and subsequent degradation, of Iκ-B (Figure 1). Vincristine, on the other hand, decreased the cytosolic p65 expression indicating p65 translocation to the nucleus and activation of NF-κB pathway. This finding is consistent with published data demonstrating that vincristine and other microtubule inhibitors activate NF-κB [24]. In the combination treatment where cells are exposed to ML120B for one hour prior to vincristine, p65 was sequestered in the cytosol comparable to levels of ML120B-alone treated cells. These findings were confirmed by immunofluorescence studies (Figure 7) and led us to hypothesize that ML120B synergizes with vincristine by preventing vincristine-induced activation of NF-κB pathway. As a transcription factor, NF-κB controls many molecules that are associated with resistance to programmed cell [3,17,18].

### Antitumor Activity of ML120B in Lymphoma-bearing SCID Mice

**Figure 8:**
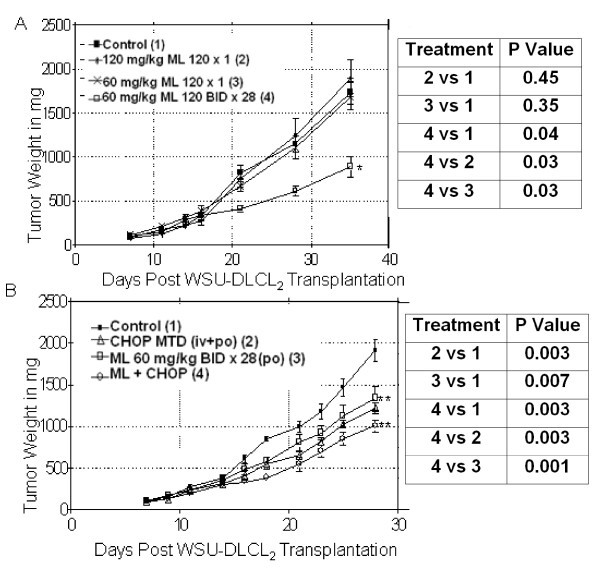
***In vivo *activity of ML120B alone, and with CHOP in WSU-DLCL2-SCID xenograft mouse model, tumor weights represent Mean and standard error of mean (SEM)**. A. Activity of ML120B as single agent: WSU-DLCL_2 _tumors were xenografted s.c. into 10 ICR SCID mice/group on day 0 and dosing was initiated on day 7. ML120B was administered p.o. 120 mg/kg one dose on day 7 (× 1), 60 mg/kg twice (BID) on day 7 (× 2), and 60 mg/kg BID for 28 days starting on day 7 (× 28). Each point represents mean tumor weight of animal in each group ± SEM. B. ML120B plus CHOP treatment: Xenografts were developed as in A and treatment started on day 7. CHOP was administered at maximum tolerated doses (MTD) as previously defined (see Material and Methods), ML120B was administered p.o. 60 mg/kg BID for 28 days (as in A), ML120B in combination with CHOP at MTD.

Finally, we determined the efficacy of ML120B in our lymphoma-bearing xenograft SCID mouse models. ML120B did not prevent WSU-FSCCL from infiltrating into the CNS in this systemic model (data not shown). It was not possible, therefore to determine its systemic efficacy since the usual cause of animal death is CNS lymphoma [25]. Conversely, ML120B delayed the growth of WSU-DLCL_2 _SC tumors. In Figure 8A, single day doses did not induce significant tumor growth delay. However, a 28-day course showed significant delay in tumor growth compared to single day doses (P = 0.03) and to control (P = 0.04). To determine whether our *in vitro *combination findings correlated *in vivo*, we compared ML120B with CHOP at its MTD. Figure 8B, shows that CHOP and ML120B significantly reduced tumor load when given alone compared to control (P = 0.003 and 0.006, respectively). ML120B: CHOP combination significantly delayed tumor growth compared to control (P = 0.003), CHOP alone (P = 0.003), and ML120B alone (0.001). This data indicate that IKK-2 inhibition potentiates conventional cytotoxic chemotherapy effect *in vivo*.

## Discussion

In this study we show that inhibition of IKK-2 by a small molecule inhibitor, ML120B, enhances the cytotoxic effect of the microtubule inhibitor, vincristine in lymphoma cells. IKK-2 inhibition leads to sequestration of p65 in the cytosol and prevention of vincristine-induced nuclear translocation. It was previously shown that NF-κB activation is involved in vincristine resistance [26]. This is believed to be due, at least in part, to the positive effect of NF-κB on cell cycle progression [27].

There are multiple approaches to target IKK-2/NF-κB pathway. Several specific IKK-2 inhibitors are under development (reviewed by Karin et. al [28]). These inhibitors have a wide range of IC_50 _in relationship to inhibiting IKK-2. For example, the IC_50 _of SPC-839, PS-1145, and BMS-345541 are 62 nM, 0.15 μM and 0.3 μM, respectively. ML120B inhibits IKK-2 at an IC_50 _of 62 nM. ML120B, in the nM range is highly specific to IKK-2, but is capable of inhibiting IKKε and other enzymes at an IC_50 _greater that 100 μM [29]. Other novel SMIs, such as GS143 suppress IκBα ubiquitination, but not IκBα phosphorylation. Thus, inhibition of NF-κB activation is as complex as the activation pathway itself with multiple sites as targets for inhibition [30].

The present study makes several key observations regarding IKK-2 as a potential therapeutic target in lymphoma. First, we demonstrated that inhibition of IKK-2 by ML120B can cause growth inhibition in a concentration- and time-dependent manner. The cause of the growth inhibition was due to the increase of cells in G0/G1 phase of the cell cycle. Our results suggest that ML120B alone acts by blocking cell growth and not via apoptosis. Second, we demonstrated that ML120B can inhibit constitutive activation of NF-κB in indolent and aggressive lymphoma cell lines in a concentration dependent manner similar to what was observed in myeloma cells [20]. These observations suggest a broad application of IKK-2 inhibition in lymphoid tumors.

Interestingly, our data shows that IKK-2 inhibition synergizes the cytotoxic effects of microtubule inhibitor, vincristine. This synergy was found at 1/100 the dose of vincristine alone required to induce comparable G2/M arrest and apoptosis (520 pM in combination with ML120B (40 μM) vs 50 nM when used alone, Figure 3A-C). Furthermore, our results suggest that the ML120B: vincristine combination induces cell cycle arrest followed by apoptosis out of G2/M. Vincristine is a microtubule depolymerizing agent. It was shown that depolymerization of microtubules activates NF-kB and induces NF-kB-dependent gene expression [31]. Our data indicate that prevention of vincristine-induced nuclear translocation of p65 and activation of NF-κB is a major mechanism of synergy between IKK-2 inhibition and vincristine. This synergy is selective since we did not observe similar interaction between IKK-2 inhibition and cyclophosphamide or doxorubicin. Cell death induced by the ML120B: vincristine combination is through the apoptosis pathway since there was evidence for caspase 3 and PARP cleavage (Figure 6). Constitutive activation of NF-κB in lymphoma and consequent activation of downstream molecules like cIAP2 [32], p21 [33], and Bcl-2 [12] increases the threshold for apoptosis. This cell survival mechanism is accentuated by exposure of cells to vincristine [24,33]. IKK-2 inhibition, by sequestering NF-κB in the cytosol and consequent down regulation of pro-survival molecules, lowers the threshold of apoptosis in response to cytotoxic agents like vincristine (Figures 3, 4, 5).

*In vivo*, we showed that ML120B: CHOP combination was well tolerated by the animals and induced higher anti-tumor efficacy compared with each agent alone in our WSU-DLCL_2_-SCID model (Figure 8). We have previously shown that genistein (30 μM) sensitizes DLCL_2 _cells to CHOP [24]. Bharti et al., have shown that curcumin, a natural inhibitor of NF-κB, may sensitize the cytotoxic effects of vincristine (50 μM) [34]. Sanda et al., showed that IKK inhibition by ACHP (10 μM) led to growth inhibition of MM cells and potentiation of vincristine cytotoxicity [35].

## Conclusion

In summary, our study shows the feasibility of inhibiting a constitutively active NF-κB pathway in lymphoma cells. Such inhibition is associated with therapeutically beneficial biological effects *in vitro *and *in vivo*. When used alone, ML120B elicited modest therapeutic gains. However, there was significant synergy with the microtubule inhibitor, vincristine. Our data indicate that approaches to NF-κB pathway inhibition are best used in combination with cytotoxic chemotherapy rather than single agents. The major future challenge is to develop a more effective IKK-2 inhibitor with lower cellular IC50 in order to make them more attractive clinically.

## Materials and methods

### Cell Culture and Reagents

The cell lines used in the study have been previously described; Follicular Lymphoma (WSU-FSCCL) [36] and Diffuse Large Cell Lymphoma (WSU-DLCL_2_) [37]; The WSU-FSCCL cell line has been karyotyped at least 4 times since our initial publication in 1993. The recent analysis in September of 2009 revealed the same chromosomal abnormalities as previously reported; 47,XY,+der(1)i(1)(q10)del(1)(q32),t(1;13)(p31;q12),del(6)(q21q27),t(8;11)(q24;q22),t(14;18)(q32;q21). The WSU-DLCL2 has been similarly karyotyped several times since its establishment in 1990. The cell line acquired an additional abnormality, add(8q24), that was detected for the first time in 1997. Since then the karyotype profile has remained stable with no further changes. The most recent karyotype in September of 2009 revealed: 48,XY,t(1;2)(p36;q37),der(3)t(3;7)(q13;p15),t(4;14)(q27;q32),+7,i(7)(p10),der(7)t(3;7)(q21;p11.2),+8,add(8)(q24),t(14;18)(q32;q21),del(15)(q26.1),del(16)(q22)[10]. Furthermore, fluorescent in situ hybridization (FISH) using LSI MYC dual color break-apart DNA probe (Vysis Inc.) revealed a deletion of the telomeric 3' region of CMYC gene most likely due to unbalanced translocation affecting the CMYC gene region. Cells were maintained in RPMI-1640 medium containing 10% heat-inactivated fetal bovine serum (FBS), 1% L-glutamine, 100 U/ml penicillin G and 100 μg/ml streptomycin and incubated at 37°C in a humidified incubator with 95%/5% CO_2_. Primary antibody specific for Actin was obtained from Santa Cruz Biotechnology, (Santa Cruz, CA). Primary antibodies specific for Caspase-3, Caspase-9, PARP, p-IκBα and IκBα were obtained from Cell Signaling, (Danvers, MA). G3PDH was obtained from Trevigen, Inc (Gaithersburg, MD). Protein concentrations were determined using the Micro BCA protein assay (Pierce Chemical Company, Rockford, IL). Cyclophosphamide monohydrate was obtained from Mead Johnson (Evansville, IN). Doxorubicin hydrochloride was obtained from Bedford Inc (SA, Australia). Vincristine was obtained from Pharma Inc. (Bloomington, MN). ML120B was synthesized by Millennium Pharmaceuticals, Inc (Cambridge, MA) and dissolved in DMSO. Concentration of DMSO in the final culture was 0.44%.

### Western Blot Analysis

Proteins obtained from cell extracts were collected 24, 48, or 72 h after single or combination treatment with the IKK-2 inhibitor (ML120B) and vincristine in lysis buffer containing protease inhibitors. Cytosolic protein extracts were prepared from control and treated cells using Nuclear/Cytosolic Fractionation Kit according to manufacturer's protocol (BioVision, Mountain View, CA). All proteins were resolved using 12% SDS-PAGE and transferred to Hybond C-extra membranes (Amersham Life Science, Arlington Heights, IL). Membranes were blocked with 5% milk in Tris buffer saline containing 0.05% Tween 20 (TBST) for 1 h at 25°C and incubated overnight at 4°C with rabbit anti-caspase 9 (H-170, Santa Cruz), rabbt anti-caspase 8 (H-134, Santa Cruz), rabbit anti-PARP (#9542, Cell Signaling), mouse anti-caspase 3 (#9668, Cell Signaling) or rabbit anti-NF-κB (H-286, Santa Cruz) in 2% Bovine serum albumin in TBST (1:1000 dilutions in BSA-TBST). Following incubation, membranes were washed with TBST and incubated with corresponding horseradish peroxidase-conjugated secondary antibody (Santa Cruz Biotechnology, Santa Cruz, CA; 1:5000 dilution in 5% milk-TBST) for 1 h at 25°C and then washed before proteins were visualized using picoglow HRP substrate (Michigan Diagnostics, LLC, Royal Oak, MI).

### Flow Cytometric Analysis of Cell Cycle and Apoptosis

Cell cycle analysis and sub G0/G1 DNA content were determined by flow cytometry using propidium iodide (PI) staining. Cells were grown in the presence or absence of ML120B or vincristine then centrifuged and washed. The cells were then fixed with 75% ice-cold ethanol overnight and stained with 50 μg of PI and analyzed. To determine DNA fragmentation (as indication of apoptosis) induced by treatment agents, we utilized standard terminal deoxynucleotidyl transferase of dUTP nick end labeling (TUNEL) assay and propidium iodide (PI) staining. The kit used in this method (ApoDirect In Situ DNA Fragmentation Assay Kit, BioVision, Mountain View, CA; Catalog #K402-50) utilizes terminal deoxynucleotidyl transferase (TdT) to catalyze incorporation of DUTP at the 3'-hydroxyl ends of the fragmented DNA. The fluorescein-labeled DNA was detected by flow cytometry (horizontal axis in Figure 4). PI staining was simultaneously used to separate cells into G0/G1, S, G2 M and sub-G0 compartments based on DNA content (vertical axis, Figure 4). The dual staining (dUTP and PI) allowed us to assign dUTP-positive cells to a cell cycle phase. In this method, it is accepted that dUTP-positive cells are considered apoptotic [38]. To confirm induction of apoptosis, we stained WSU-FSCCL cells with 7-AAD as previously published from our laboratory [39]. All flow cytometry analysis of cells was done on FACScan (Becton-Dickinson, San Jose, CA).

### Fluorescence Microscopy

WSU-FSCCL cells, treated and untreated, were harvested, washed once with PBS and fixed for 10 min with 3.7% formaldehyde in PBS. All procedures were carried out at room temperature. Following fixation, cells were washed 3 times with PBS, blocked for 45 min with 0.5% BSA in PBS and then incubated for 3 hr in 200 μl PBS containing 0.1% saponin (PBS-S), 1 μg/ml each of two primary antibodies, mouse anti-human NF-κBp65 and rabbit anti-tubulin. After incubation with primary antibodies, cells were carefully washed 3 times with PBS-S and then resuspended in PBS-S containing 5% goat sera and 10 μg/ml each of two fluorescently-labeled secondary antibodies and DAPI (10 μg/ml) for nuclear staining, if used. Cells were incubated for 1 hour, washed X3 with PBS-S and then fixed for 1 min with 3.7% formaldehyde. Following the final fixation, cells were washed 3 times with PBS containing no saponin. Cell suspensions were mounted on 1% gelatin-coated slide, dried, sealed with coverslips and visualized using an Olympus BX40 microscope equipped with laser light and fluorescence filter cubes for UV, green and red fluorescence. Visual recordings were captured separately using an RT-Spot Color Camera (Diagnostic Instruments, Inc, Sterling Heights, MI) and merged using Super Spot software (Diagnostic Instruments) to complete the overlay and final pictures.

All primary antibodies were purchased from Cell Signaling Technologies (Danvers, MA). Slow Fade Light, DAPI and Alexa Fluor 488 (green) and Alexa Fluor 568 (red) fluorescently labeled secondary antibodies were purchased from Molecular Probes (a division of InVitrogen, Carlsbad, CA).

#### Establishment and Propagation of Xenografts

3-4 week old female ICR mice with severe combined immune deficiency (SCID) were purchased from Taconic Farms (Germantown, NY). Animals were housed in special protective environment and left to adapt for few days before beginning the experiments. To initiate the WSU-DLCL_2_-SCID xenografts, (5-10) × 10^6 ^WSU-DLCL_2 _cells in serum-free RPMI 1640 medium were injected subcutaneously (SC) in the flank areas of each animal. Palpable tumors were detected by clinical examination in about 2 weeks. When tumor weight reached 1000-1500 mg, animals were euthanized; tumors dissected out, placed in RPMI 1640 medium in sterile environment and minced into small fragments (20-30 mg each). To propagate the xenografts, tumor fragments were implanted SC, using a trocar, into flanks of 3-4 week old female ICR-SCID mice. Forty animals were implanted with WSU-DLCL_2 _tumors for the single agent (ML120B alone) experiment and forty for the combination study (CHOP plus ML120B). The WSU-FSCCL-SCID is a systemic model which is established by injecting 10^7 ^WSU-FSCCL cells in serum-free medium intravenously via tail vein of ICR-SCID mice. The growth pattern and assessment of response of this model to ML120B were the same as previously published from our laboratory [25].

#### Efficacy Trial Design

WSU-DLCL_2 _tumor-bearing animals were randomly assigned to control or one of 3 treatment dose/schedules of ML120B; 10 animals in each group. Therapy was started one week after tumor implantation. Group 1 received one dose of ML120B at 120 mg/kg. Group 2 received 60 mg/kg twice (every 12 hours). Group 3 received 60 mg/kg twice a day for 28 days. All treatments were given through oral gavage. ML120B compound was dissolved in 5% (hydroxypropyl) methylcellulose. Control group animals received vehicle alone. CHOP MTD in SCID mice was previously determined in our laboratory [21] for one injection (i.e.40 mg/kg, i.v. cyclophosphamide; 3.3 mg/kg,i.v. doxorubicin; 0.5 mg/kg,i.v. vincristine; and 0.2 mg/kg orally prednisone every day for 5 days). Animals were monitored 3 times per week for signs of toxicity, weight changes and tumor measurements. They were euthanized to avoid discomfort if the tumor burden reached ~2000 mg (approximately 10% of the body weight). All animal experiments were done according to protocols approved by the Animal Investigation Committee (AIC) of Wayne State University.

### Statistical Analysis

Statistical significance of drug-treated versus control measurements was determined by the student t-test. The interaction between ML120B and vincristine was analyzed using Calcusyn V2 software program to determine if the combinations were synergistic. Calcusyn is based on the Chou-Talalay method [40], which calculates a combinational index (CI) to indicate synergistic effects where CI < 0.9, is considered synergistic. Survival functions were estimated using the Kaplan-Meier method and compared by the log-rank test. P-values <0.05 were considered statistically significant.

All statistical analyses were evaluated using GraphPad Prism 4 (San Diego, CA).

## Competing interests

The authors declare that they have no competing interests.

## Authors' contributions

AK had overall supervision of the project, data analysis and manuscript writing. AAA did technical conduct of in vitro experiments and manuscript writing. AA did the animal experiments. AS did p65 blots and assisted in immunofluorescence experiments and experimental design. PS provided the ML120B IKK-2 inhibitor and data on binding to its target; critiqued manuscript. AM re-characterized the WSU-FSCCL and WSU-DLCL2 cell lines used in the study by cytogenetics and FISH; compared data with previous genetic profiles of same cell lines. FB did the immunofluorescence experiments; participated in manuscript writing and critique. RM supervised Dr. Arnold; Animal data analysis; review and critique of the manuscript.

All authors read and approved the manuscript.
